# Probiotic Effector Compounds: Current Knowledge and Future Perspectives

**DOI:** 10.3389/fmicb.2021.655705

**Published:** 2021-03-03

**Authors:** Eric Banan-Mwine Daliri, Fred Kwame Ofosu, Chen Xiuqin, Ramachandran Chelliah, Deog-Hwan Oh

**Affiliations:** Department of Food Science and Biotechnology, College of Agriculture and Life Science, Kangwon National University, Chuncheon, South Korea

**Keywords:** microbiota, gut barrier functions, immune system, cholesterol reduction, nervous system

## Abstract

Understanding the mechanism behind probiotic action will enable a rational selection of probiotics, increase the chances of success in clinical studies and make it easy to substantiate health claims. However, most probiotic studies over the years have rather focused on the effects of probiotics in health and disease, whereas little is known about the specific molecules that trigger effects in hosts. This makes it difficult to describe the detailed mechanism by which a given probiotic functions. Probiotics communicate with their hosts through molecular signaling. Meanwhile, since the molecules produced by probiotics under *in vitro* conditions may differ from those produced *in vivo*, *in vitro* mechanistic studies would have to be conducted under conditions that mimic gastrointestinal conditions as much as possible. The ideal situation would, however, be to carry out well-designed clinical trials in humans (or the target animal) using adequate quantities of the suspected probiotic molecule(s) or adequate quantities of isogenic knock-out or knock-in probiotic mutants. In this review, we discuss our current knowledge about probiotic bacteria and yeast molecules that are involved in molecular signaling with the host. We also discuss the challenges and future perspectives in the search for probiotic effector molecules.

## Introduction

**Figure 1:**
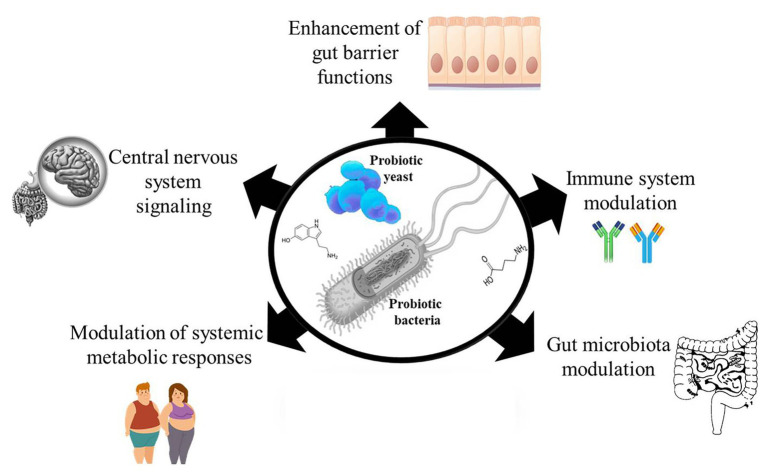
Probiotic bacteria and yeast possess and produce many effector molecules that interact with the host to elicit their observed effects. The effector molecules can affect host gut epithelial barrier functions, modulate host immune system, modulate the host’s gut microbiota, modulate the host’s metabolic responses, and affect the host’s central nervous system.

Probiotics are live microorganisms which when administered in adequate quantities provide a beneficial effect to the host (Hill et al., 2014). Over the years, many randomized clinical studies have reported the health benefits of probiotic consumption (Chugh et al., 2020; Tao et al., 2020; Tenorio-Jiménez et al., 2020), yet monitoring targeted health benefits of administered probiotic have rather been difficult to establish. This is because it would require an understanding of the possible metabolic activities of the administered probiotic that distinguishes it from the indigenous microbes in the host that could be eliciting similar effects. The limited knowledge about the mechanisms of probiotic action makes it challenging to rationally select probiotic strains for targeted interventions and also makes reproducibility of results difficult. Meanwhile, a number of mechanisms have been proposed to explain the mode of action behind the health effects of probiotics, such as enhancing gut epithelia barrier functions (Liu et al., 2016), modulating the immune system (Milajerdi et al., 2020; Zhao et al., 2020), modulating the gut microbiota (Botelho et al., 2020; López-Moreno et al., 2020), modulating systemic metabolic responses (van Baarlen et al., 2011; Belguesmia et al., 2016), and signaling host central nervous system (Kong et al., 2020; Figure 1). Since probiotic and host cell interactions are mediated by effector molecules, we discuss our current knowledge on probiotic bioactive molecules that have impact on the host in this review. We also discuss the challenges and future perspectives in the search for probiotic effector molecules.

## Probiotic Effector Molecules That Affect Gut Barrier Functions

Current scientific evidence show that a disruption of gut epithelial barrier function is important in the pathogenesis of many diseases such as inflammatory bowel disease (IBD; Lee et al., 2018; Soroosh et al., 2019), irritable bowel syndrome (Lee et al., 2020), diabetes (De Kort et al., 2011), and several other diseases. Gut barrier integrity is maintained by tight junction proteins, such as claudins, Zona occludin-1, and occludin, and their levels are significantly reduced during some disease conditions (Lee et al., 2020). This makes the gut epithelium permeable to microbial ligands and harmful metabolites leading to systemic inflammatory responses (Singh et al., 2019). The ability of probiotic effector molecules to protect gut barrier functions has long been reported (Miyauchi et al., 2009; Karczewski et al., 2010; Laval et al., 2015; Martín et al., 2019). Some of the molecules are soluble and therefore are secreted by the bacteria (or fungi) while others are cell wall bound (Delgado et al., 2020). *Lactobacillus rhamnosus* GG has been shown to secrete protein p40, which stimulates ADAM17 activation and heparin binding-epidermal growth factor (HB-EGF) release. This results in EGF receptor transactivation, apoptosis prevention, and intestinal epithelial function preservation (Yan et al., 2013). Another soluble protein, p75 secreted by both *L. rhamnosus* and *L. casei* is known to stimulate EGF receptor activation to prevent apoptosis in intestinal epithelial cells (Bäuerl et al., 2010). More recently, *L. rhamnosus* GG was shown to secrete protein HM0539, which protects gut barrier functions by promoting the expression of tight junction protein Zona occludin-1 and occluding. The protein also stimulates mucin secretion in intestinal cells (Gao et al., 2019). Meanwhile, the exact molecular mechanism by which HM0539 protects intestinal cells from injury remains to be established. Previous studies have shown that the soluble protein known as TcpC protein produced by *Escherichia coli* Nissle 1917 can induce protein kinase C-ζ and extracellular-signal-regulated kinase 1/2 phosphorylation to increase the formation of claudin-14, and this could account for the use of the probiotic for gastrointestinal therapy (Hering et al., 2014). *Escherichia coli* Nissle 1917 also produces 3-hydroxyoctadecanoic acid, which antagonizes peroxisome proliferator activated receptor gamma (PPARγ) to reduce inflammation (Pujo et al., 2020). In addition, probiotics and several other lactic acid bacteria are known to produce conjugated linoleic acid (CLA; Wang et al., 2016), which can upregulate the transcription of E-cadherin 1, claudin-3, ZO-1, and occludin in the gut to protect gut barrier functions (Murphy et al., 2007; Chen et al., 2019). Probiotic CLA can increase the expression of catalase, superoxide dismutase, and glutathione peroxidase, which reduce oxidative stress in colonocytes (Qi et al., 2018; Chen et al., 2019). More so, CLA increases the expression and activity of PPARγ in the gut to inhibit inflammation (Hontecillas et al., 2002). Some structural components, such as pili, of many probiotic bacteria have been shown to play important roles in gut epithelial functions. For instance, the tight adhesion pili of *Bifidobacterium breve* UCC2003 have been reported to stimulate the proliferation of gut epithelial cells by producing a TadE pseudopilin (O’Connell Motherway et al., 2019). Also, *L. plantarum* CGMCC 1258 micro integral membrane protein (MIMP) was found to promote the expression of tight junction proteins, such as JAM-1, claudin-1, and occludin, during tight junctional injury (Yin et al., 2018). Meanwhile, the exact mechanism by which MIMPs promotes the upregulation of tight junction proteins remain unestablished.

## Probiotic Effector Molecules That Stimulate the Immune System

Many studies have reported the ability of the probiotics to contribute to the maturation of the immune system. It has however been shown that lipopolysaccharide of Gram negative probiotics are responsible for their strong induction of IL-10 in peripheral blood mononuclear cells (Kandasamy et al., 2017). The IL-10 produced contributes to the induction of IgA antibodies at mucosal sites by enhancing isotypic commutation (Cerutti and Rescigno, 2008), and this may account for why Gram negative probiotics induce a stronger antibody response than Gram positive probiotics. This was demonstrated when *E. coli* Nissle 1917 colonized pigs showed a higher gut IgA response relative to *L. rhamnosus* GG colonization (Kandasamy et al., 2016). The anti-inflammatory ability of *L. plantarum* in acute colitis mice (Emília et al., 2018) has at least in part been attributed to the presence of a small domain of the surface layer protein of the bacterium. These surface layer proteins have been shown to bind to a mannose receptor in gut epithelial cells to prevent p38 MAPK phosphorylation by inhibiting the Toll-like receptor (TLR) 5 pathway (Liu et al., 2012) during inflammatory conditions. This suppresses the expression of inflammatory cytokines (IFN-γ, IL-17, and IL-23) and upregulates IL-4 and IL-10 production in the cells (Yin et al., 2018). Another study has shown that sortase-dependent protein on the cell surface of *L. plantarum* can actively attenuate the NF-kB pathway thereby suppressing inflammation (Marco et al., 2010; Remus, 2012). It is known that different immune-stimulating probiotic strains may however have different cell wall molecules that interact with host immune receptors (Bron et al., 2013; Lee et al., 2013). For instance, the immune stimulating ability of *L. plantarum* K8 has been partly attributed to the lipotechoic acid (LTA) in its cell wall, which is able to regulate mitogen-activated protein kinase phosphorylation and nuclear factor activation and causes a reduction in IL-8 production in injured intestinal cells (Kim et al., 2017). Meanwhile, LTA from *L. rhamnosus* GG, *L. sakei*, and *L. delbrueckii* did not stimulate IL-8 production under similar conditions (Kim et al., 2017). In probiotic yeast, *Saccharomyces cerevisiae* cell wall contains β-glucans, which may induce monocyte reprograming *via* a dectin-1/Raf-1 pathway to enhance cytokine production for protection against *Candida albicans* infection (Quintin et al., 2012). Similarly, *S. cerevisiae* chitin has been shown to increase host immune system resistance to *C. albicans* infection by modulating the production of pro‐ and anti-inflammatory cytokines (Rizzetto et al., 2016). Even the spores of *S. cerevisiae* contain high amounts of chitin that can trigger inflammatory IL-17 responses in hosts (Rizzetto et al., 2010).

## Probiotic Effector Molecules That Modulate Host Central Nervous System

**Figure 2:**
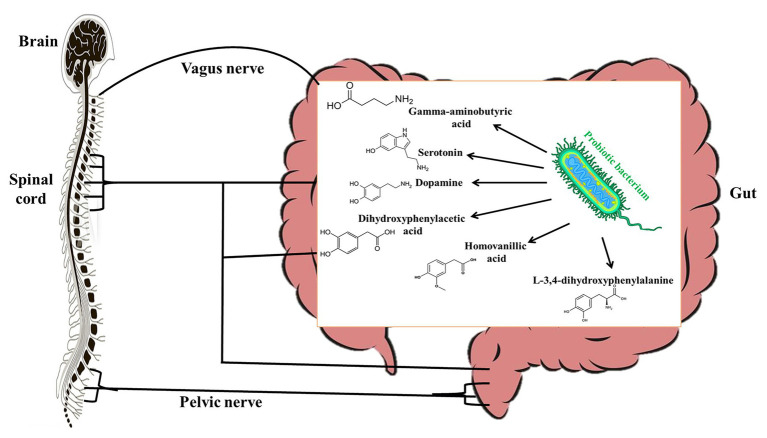
Probiotic bacteria produce gamma-aminobutyric acid (GABA), serotonine, dopamine, L-3,4-dihydroxyphenylalanine (L-DOPA), dihydroxyphenylacetic acid (DOPAC), and homovanillic acid (HVA) that modulate the host central nervous system.

Studies over the years have shown evidence (though mostly indirect) that there is a strong communication between the gut microbiota and the central nervous system and it is mediated by the vagal nerve (Cryan and Dinan, 2012). For this reason, the effects of probiotics on the central nervous system have been studied extensively. However, only several studies have studied the molecular mechanisms by which probiotics affect the central nervous system. Many probiotic bacteria including *L. plantarum*, *L. brevis*, *L. rhamnosus*, and *Bifidobacterium bifidum* have been shown to produce significant amounts of gamma-aminobutyric acid (GABA) *in vitro* (Li et al., 2011; Diez-Gutiérrez et al., 2020). It is known that GABA in circulation may act as an autocrine, local paracrine, or gastrointestinal hormone that exerts both stimulatory and inhibitory effects over enteric neuronal activity depending on the type of GABA receptor stimulated (Hardcastle et al., 1991). Interestingly, Koussoulas et al. (2018) have shown that exogenous GABA can bind to GABA_A_, GABA_B_, and GABA_c_ receptors to induce calcium [Ca^2+^]_i_ release by myenteric ganglia. Since glial Ca^2+^ signaling is a mechanism for integration within glial symplasm and between glial-neuronal circuits (Verkhratsky, 2006), it stands to reason that GABA produced by probiotics could be an effector molecule by which certain probiotics interact with host central nervous system. This could therefore account for the ability of *L. rhamnosus* JB-1 to alter stress-related disorders *via* the vagus nerves (Bravo et al., 2011). Other studies have shown that *L. helveticus* 100ash, *L. helveticus* NK-1, *L. casei* K3III_24_, and *L. delbrueckii* subsp. *bulgaricus* produce significant amounts of GABA, L-3,4-dihydroxyphenylalanine (L-DOPA), dopamine, dihydroxyphenylacetic acid (DOPAC), homovanillic acid (HVA), and serotonin (Figure 2) when cultured in milk-containing media (Oleskin et al., 2014). L-DOPA can be transported from the gut through the blood to the brain, where it is converted to dopamine, a neurotransmitter (Disdier and Stonestreet, 2019). Dopamine can be metabolized into DOPAC, which can be degraded to HVA. In the central nervous system, serotonin plays a role in regulating emotions, sleep, and stress (De Deurwaerdère and Di Giovanni, 2020) by influencing the hypothalamic-pituitary-adrenal axis. Though it is likely that probiotics that produce these biogenic amines may have central nervous system modulatory effects, the effector molecules would have to be produced in adequate quantities in the gut of the host to elicit the desired effects. Although some studies have suggested that probiotics may improve diseases associated with the central nervous system by enhancing the production of free tryptophan, which may promote serotonin availability (since serotonin is synthesized from tryptophan; Desbonnet et al., 2008), it is not known whether probiotic tryptophan is used for serotonin synthesis (Daliri et al., 2016).

## Probiotic Effector Molecules That Modulate Host Microbiota

**Figure 3:**
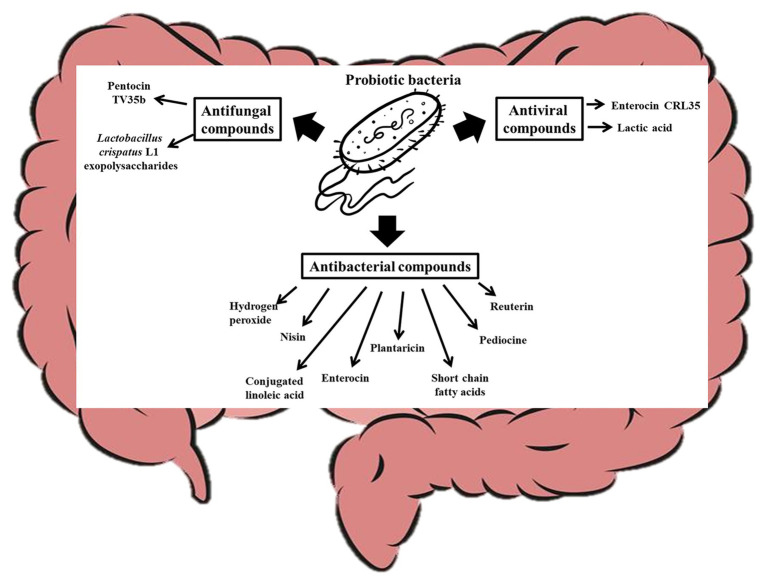
Probiotic bacteria modulate the gut microbiota by producing antifungal, antiviral, and antibacterial compounds that affect the overall gut microbial structure of the host.

The human body is made of mammalian cells, archaea (Kim et al., 2020), bacteria, viruses, and fungi co-existing in a symbiotic relationship (Daliri et al., 2020). Although host microbes could vary from one person to another (Brooks et al., 2018), recent studies have shown that all these microorganisms in the human body could have impacts on host health and disease (Daliri et al., 2018; Reitmeier et al., 2020) and that probiotic consumption can significantly modulate the host microbiome (Alcon-Giner et al., 2020). In fact, probiotics in food can survive, adapt, and become an established part of the gut microbiome (Pasolli et al., 2020) and have significant influence on the gut microbiome. The mechanisms by which probiotics modulate host microbiome include their effects on the function and composition of the host commensal bacteria and yeast. It is known that probiotics can produce antimicrobial compounds that suppress (O’Shea et al., 2012) or promote (Chen et al., 2019) the growth of certain microorganisms in the gut as shown in Figure 3. *Lactococcus lactis* for instance produces nisin (a bacteriocin), which can permeate the cell membrane of pathogenic Gram positive-bacteria and bind to Lipid II to inhibit cell wall synthesis (Wiedemann et al., 2001; Breukink and de Kruijff, 2006). Other probiotics such as *Pediococcus acidilactici* produce pediocine-like bacteriocins, which can bind to mannose phosphotransferase on the cytoplasmic membrane of pathogenic bacteria and penetrate the lipid bilayer resulting in pore formation in the cell (Balandin et al., 2019). This compromise in the cell wall integrity makes the cell extremely vulnerable to the harsh environmental conditions within which the cell is found and can lead to death. Similarly, Plantaricin LPL-1 produced by *L. plantarum* LPL-1 binds to a receptor on *Listeria monocytogenes* cell wall, perforate the cell membrane through hydrophobic interactions, and accumulate in the cell membrane by electrostatic interactions. The perforation results in ion leakage and loss of proton motive force, which eventually result in cell death (Wang et al., 2019b). Plantaricins do not only inactivate Gram-positive bacteria, but also Gram-negative bacteria (Zhang et al., 2013; Yang et al., 2019). Wang et al. (2020) showed that Plantaricin BM-1 can inhibit *E. coli* by acting on the surface of the cell wall to cause cell rapture. The bacteriocin could also permeate and interact with the cell membrane to lead to cell death. A popular probiotic, *Lactobacillus reuteri*, is known to produce reuterin, which suppresses the expression of *Clostridioides difficile* exotoxins TcdA and TcdB by inducing reactive oxygen species shifts in carbon metabolism, which may alter gene expression in the pathogen (Engevik et al., 2020). The impaired metabolism of the pathogens decreases their ability to compete for nutrients and eventually result in cell death (Engevik et al., 2020). Certain bacteriocins can inhibit viruses and yeast growth. For instance, it has been shown that Enterocin CRL35 from *Enterococcus faecium* CRL35 can bind and block the late stages of herpes simplex virus types (HSV) 1 and 2 replication (Wachsman et al., 1999, 2003) while Pentocin TV35b produced by *Lactobacillus pentosus* TV35b can inhibit the survival of *C. albicans* (an opportunistic fungus; Okkers et al., 1999). Exopolysaccharides produced by *Lactobacillus crispatus* L1 also reduces *C. albicans* adhesion to epithelial cells (Donnarumma et al., 2014) thereby decreasing their tendency to invade host cells. *Lactobacillus acidophilus* La-5 produces CLA (Macouzet et al., 2009), which can repress virulence genes such as *eaeA* (an enterohaemorrhagic *E. coli* invasion lipoprotein gene) and *invH* (a *Staphylococcus typhimirium* invasion lipoprotein gene) thereby reducing the ability of these pathogens to attach to the host cells (Peng et al., 2018). Also, probiotic CLA can competitively bind to INT-407 cell surface receptor-like molecules preventing enteric pathogens from binding to the gut (Peng et al., 2018). Consumption of probiotic CLA favored an increase in the levels of *Bifidobacterium* and *Odoribacter* while reducing the levels of Bacteroides in the gut (Chen et al., 2019) through an unidentified mechanism. Other probiotics, such as *L. brevis* CD2, *L. salivarius* FV2, and *L. plantarum* FV9, produce lactic acid which impair Herpes simplex virus 2 (HSV-2) protein synthesis and viral replication (Conti et al., 2009), while bacteria including *L. paracasei* inhibit vesicular stomatitis virus by direct binding with them (Botić et al., 2007). Bacteria with strong mucosal binding abilities, such as *L. brevis*, CD2 compete with HSV-2 for binding sites thereby preventing the fusion of viral envelope with host cell surface (Conti et al., 2009). Short chain fatty acids (SCFAs), such as butyric acid, produced by probiotics can induce gut epithelia secretion of cathelicidin peptides, which inactivate pathogens such as *Shigella* (Schauber et al., 2003; Raqib et al., 2006; Termén et al., 2008; Campbell et al., 2012). Some probiotics, including *B. bifidum* (Kawasaki et al., 2009) and *Lactobacillus johnsonii* (Pridmore et al., 2008), are known to produce hydrogen peroxide, which can react with O_2_^−^ and/or iron in pathogenic cells to form toxic hydroxyl radicals which results in cell death (Clifford and Repine, 1982). A probiotic *S. cerevisiae* isolated from Koumiss has been shown to inhibit *E. coli* by producing citric acid and propionic acid, which disintegrate the cell membrane and increase cell permeability (Chen et al., 2017). Certain *S. cerevisiae* strains have been shown to produce killer toxin KHS which inhibits the growth of pathogenic bacteria and yeast (Goto et al., 1991; Younis et al., 2017). These killer toxins inhibit β-glucan synthesis in the cell walls (Muccilli et al., 2013), inhibit DNA synthesis (Klassen and Meinhardt, 2005), cleaves tRNA (Klassen et al., 2008), blocks calcium ion uptake (Brown, 2011), and cause ion leakage from the cytoplasm (Santos et al., 2007) of sensitive cells. Another study showed that chitin from *S. cerevisiae* can train host immune system to kill *S. aureus*, *E. coli*, and *C. albicans* (Rizzetto et al., 2016), and this shows how yeast molecules can play critical roles in gut microbiota modulation. Many pathogenic bacteria target glycosaminoglycans for attachment and infection of the host (Kawai et al., 2018). Quite recently, it has been shown that probiotics, such as *Enterococcus faecium*, H57 prevent pathogen adhesion to the gut by degrading GAG (which are major component of extracellular matrix in animals) using KduI and KduD enzymes (Kawai et al., 2018). The probiotic molecules discussed in this section could at least contribute to the gut modulatory ability of probiotic yeast (Adel et al., 2017; Villar-García et al., 2017) and bacteria (Kong et al., 2019; Liu et al., 2020).

## Probiotic Effector Molecules That Affect Cholesterol-Lowering

Hypercholesterolemia is a metabolic disorder marked by abnormally high levels of cholesterol in the cells and blood (Wang et al., 2019a). Since high blood cholesterol levels have been strongly associated with an increased risk of coronary heart diseases, several strategies including probiotic consumption have been used to reduce the levels of cholesterol in hypercholesterolemic patients (Jones et al., 2012). However, the mechanism behind the hypocholesterolemic ability of probiotics remains elusive. Yet one of the most reported mechanism has been attributed to the ability of some probiotics to produce bile salt hydrolase (BSH; Jones et al., 2012; Wang et al., 2019a; Huang et al., 2020). Many probiotic bacteria, such as *L. plantarum* WCFS1, *L. plantarum* TH1, and *Bifidobacteria longum* SBT2928, produce BSH (Ishimwe et al., 2015), which can hydrolyze conjugated bile salts, such as taurine-conjugated bile salts and glycine-conjugated bile salts, to release primary bile acids (Li et al., 2020). The resulting deconjugated bile acids are less soluble (Hofmann and Mysels, 1992; Degirolamo et al., 2014), and hence are less reabsorbed into circulation but excreted through feces. Bacteria, such as *L. acidophilus*, *L. bulgaricus*, and *L. casei* ATCC 393, possess intracellular and extracellular cholesterol reductases, which effectively convert cholesterol to coprostanol (Lye et al., 2010). β-glucan from some probiotic lactobacilli (London et al., 2014) and *S. cerevisiae* (Kusmiati and Dhewantara, 2016) have been reported to reduce serum cholesterol by binding to enteral bile acids to promote their excretion in feces (Sima et al., 2018). Also, probiotic SCFAs, such as butyrate, may inhibit cholesterol biosynthesis by inhibiting DL-3-hydroxy-3-methyl-glutaryl-CoA reductase, which is the enzyme that catalyzes the rate-limiting step of cholesterol biosynthesis (Marcil et al., 2003). Other studies have shown that butyrate significantly induces ATP-binding cassette sub-family A member 1 (ABCA1) *via* specificity protein 1 (Sp1) in macrophages (Du et al., 2020). ABCA1 is responsible for maintaining lipid homeostasis by regulating cellular cholesterol (He et al., 2020). This may account for the ability of SCFAs to significantly decrease the rate of hepatic and mucosal cholesterol biosynthesis (Hara et al., 1999; Du et al., 2020). Lee et al. (2010) have shown that catabolite control protein A (a membrane associated protein) plays a major role in the ability of *L. acidophilus* A4 to reduce serum cholesterol. Wild *L. acidophilus* A4 strains effectively reduced serum cholesterol levels while catabolite control protein A (*ccpA*) mutants had significantly reduced cholesterol reducing abilities. Although the direct effect of the protein on cholesterol reduction remains unclear, the *ccpA* gene is known to strongly regulate important cell functions including lipid metabolism, cell envelope biogenesis, carbohydrate transport and metabolism, outer membrane and intracellular cargo trafficking (Zomer et al., 2007), and hence affect cholesterol metabolism. Certain gut bacteria, such as *Eubacterium coprostanoligenes*, have been identified to possess *ismA* genes, which express 3β-hydroxysteroid dehydrogenase for converting cholesterol to cholestenone, to coprostanol, and then to coprostanone (Kenny et al., 2020). The bacterium is a good candidate of next generation probiotics as it effectively decreases total cholesterol levels in foods (Madden et al., 1999) and animals (Li et al., 1995, 1996, 1998). However, the *ismA* gene has not been reported in known probiotics yet.

## Challenges and Future Perspectives on Probiotic Effector Molecules

Over the years, server studies have successfully identified as soluble molecules of probiotics in media by using metabolomics approaches (Kahouli et al., 2015; Usta-Gorgun and Yilmaz-Ersan, 2020). After recovery, the molecules are applied in *in vitro* and *in vivo* studies to confirm their effects (Wiedemann et al., 2001; Li et al., 2020). For membrane bound molecules, however, the cells are usually broken and the components separated (Urner et al., 2020) and tested for activity. Meanwhile, the metabolic activities of microbes may change when their biological niches are changed (Franzosa et al., 2014), and so the bioactive molecule identified *in vitro* may not be the cause or the only possible cause of the physiological effect observed in the host after probiotic administration. This has been observed in studies in which knocking out genes suspected to express certain active molecules did not completely attenuate the physiological effects of the bacterium (Lee et al., 2010). Therefore, future studies may have to consider carrying out *in vitro* tests of the production of probiotic active molecules with gastrointestinal effects under simulated gastrointestinal conditions (mixture of enzymes, acids, salts, mucus, etc.). Such studies may also have to consider the influence of disease conditions on the immune system, host antimicrobial proteins, and gut microbial competition on the probiotic in other to ascertain what genes (and bioactive compounds) are really triggered (and produced) in consumed probiotics. A more plausible way of assessing the probable effects of these stressors on a consumed probiotic would be to collect the microbe from gut samples (or vagina) after the expected physiological effect has occurred and subjected to transcriptomics to ascertain if the mRNA of the suspected bioactive molecule (in case of a protein or peptide) is actively transcribed under those conditions.

For a better understanding of which probiotic molecules trigger a given effect, there is the need to carry out well-designed clinical trials in humans using adequate quantities of the isolated bioactive molecules from probiotics or adequate quantities of isogenic knock-out or knock-in probiotic mutants. This is essential because animals and humans are different and so extrapolating results from animal studies may not always be correct. Meanwhile, such a study would face several ethical and technical hurdles as it involves humans and will have to establish the probiotic strain’s potency, effective dose, targeted host response, targeted host site, and other important parameters.

## Author Contributions

ED conceived, designed, and wrote the manuscript. FO, CX, and RC revised and made corrections. D-HO approved the manuscript and provided funding. All authors contributed to the article and approved the submitted version.

### Conflict of Interest

The authors declare that the research was conducted in the absence of any commercial or financial relationships that could be construed as a potential conflict of interest.
